# Recombination and Evolution of Duplicate Control Regions in the Mitochondrial Genome of the Asian Big-Headed Turtle, *Platysternon megacephalum*

**DOI:** 10.1371/journal.pone.0082854

**Published:** 2013-12-18

**Authors:** Chenfei Zheng, Liuwang Nie, Jue Wang, Huaxing Zhou, Huazhen Hou, Hao Wang, Juanjuan Liu

**Affiliations:** Provincial Key Lab of the Conservation and Exploitation Research of Biological Resources in Anhui, Anhui Normal University, Wuhu, Anhui, P.R. China; Beijing Institute of Genomics, China

## Abstract

Complete mitochondrial (mt) genome sequences with duplicate control regions (CRs) have been detected in various animal species. In Testudines, duplicate mtCRs have been reported in the mtDNA of the Asian big-headed turtle, *Platysternon megacephalum*, which has three living subspecies. However, the evolutionary pattern of these CRs remains unclear. In this study, we report the completed sequences of duplicate CRs from 20 individuals belonging to three subspecies of this turtle and discuss the micro-evolutionary analysis of the evolution of duplicate CRs. Genetic distances calculated with MEGA 4.1 using the complete duplicate CR sequences revealed that within turtle subspecies, genetic distances between orthologous copies from different individuals were 0.63% for CR1 and 1.2% for CR2app:addword:respectively, and the average distance between paralogous copies of CR1 and CR2 was 4.8%. Phylogenetic relationships were reconstructed from the CR sequences, excluding the variable number of tandem repeats (VNTRs) at the 3′ end using three methods: neighbor-joining, maximum likelihood algorithm, and Bayesian inference. These data show that any two CRs within individuals were more genetically distant from orthologous genes in different individuals within the same subspecies. This suggests independent evolution of the two mtCRs within each *P. megacephalum* subspecies. Reconstruction of separate phylogenetic trees using different CR components (TAS, CD, CSB, and VNTRs) suggested the role of recombination in the evolution of duplicate CRs. Consequently, recombination events were detected using RDP software with break points at ≈290 bp and ≈1,080 bp. Based on these results, we hypothesize that duplicate CRs in *P. megacephalum* originated from heterological ancestral recombination of mtDNA. Subsequent recombination could have resulted in homogenization during independent evolutionary events, thus maintaining the functions of duplicate CRs in the mtDNA of *P. megacephalum*.

## Introduction

Gene content, genomic architecture and gene strand asymmetry have all been reported to be stable in the mitochondrial (mt) genomes of deuterostomes, particularly the vertebrates, according to the genome stabilization theory [1]. However, as more mt genomes are sequenced, genetic variations in the control regions (CRs) have been detected in many different species, including sea cucumbers, arthropods, ostracods, fish, frogs, turtles, snakes, lizards, and birds [2]–[11]. Because control regions are large non-coding regions (LNR) that may participate in mitochondrial genome replication and regulation of transcription, genetic variances such as duplication or degeneration occurring within CRs have significant evolutionary value in the mitochondria [3]. Although in most animals, mitochondrial genomes usually contain only one CR, duplication of mitochondrial CR does occur in more species than previously thought [12]. Even triplicate CRs have been detected in the velvet worm, *Metaperipatus inae* [13].

Duplicate CRs with highly similar sequences are proposed to have evolved in a concerted fashion [3], [4], [14]–[16]. Despite the increasing reports of concerted evolution of CRs, contradictory data have been reported in the mt genome of the *Amazona* parrot, albatross, and three related sea birds belonging to the family Sulidae [15]–[17]. Eberhard documented the concerted evolution of CR within *Amazona* parrots and reported that both paralogous copies of duplicate CRs in an individual were more closely related to each other than orthologous copies of same CR in different individuals [15]. However, three exceptions to this discovery have been reported within subspecies, *Amazona ochrocephala*. The orthologous copies of CR1 and CR2 from different individuals are genetically closer to each other than to the duplicate copies (CR1 and CR2) within the same individuals. This could be attributed to independent evolution of the two copies after an ancient duplication event although the mechanism behind this is not clearly understood. Contrasting evolutionary patterns have been reported in the albatross, *Thalassarche albatrosses*, where one part of CRs evolved independently while the other showed concerted evolution [16]. Variation in duplicate CRs was detected only in two 327 bp fragments, which are referred in this study as F1 (CR1) and F2 (CR2) in five *Thalassarche* species. Conflicting phylogenetic signals were observed when different parts of these fragments were studied. The 110 bp sequence in both F1 and F2 appear to have evolved independently, but the remaining 217 bp indicate concerted evolution. Similar patterns were detected in sea birds [17]. The 51 bp at the 5′ end of the sea birds' CRs exhibited independent evolutionary signals but the duplicate CRs evolved concertedly as a whole. These conflicting evolutionary patterns of CR duplication warrant further investigation to clarify the phylogenetic relationships by investigating multiple recombination sites [16], [17].

Thus far, gene rearrangement events in mt genomes of Testudines have been discovered in only two turtles: the pancake turtle, *Malacochersus tornieri*, and the Asian big-headed turtle, *Platysternon megacephalum* [7], [8], [18]. The *P. megacephalum* is the sole living representative species of the Platyternon family and has an mt genome containing a segment with six-genes rearrangement (*tRNA^His^/tRNA^Ser^/tRNA^Leu^/nad 5* and *tRNA^Thr^/tRNA^Pro^*) and duplicate CRs [7], [8]. The evolution of CR duplication is unclear in *P. megacephalum*. To fill this lacuna, we collected 20 individuals belonging to three subspecies of the Platyternon family and determined the evolutionary patterns of the duplicate CRs in *P. megacephalum*. Finally, we discuss the possible mechanisms by which the duplicate CRs may have originated and evolved in *P. megacephalum* mtDNA.

## Materials and Methods

### Ethics statement

AH1, AH2, AH3 individuals were collected from Mount Huangshan in Anhui Province. Individual Z2 was collected from Mount Huangshan that is adjacent to Zhejiang Province. Specimens from Fujian Province were provided by the Museum of Fujian Normal University. Individuals from Guangdong, Guangxi, and Yunnan Provinces were provided by Kunming Institute of Zoology, CAS, Yunnan Province.

Procedures involving animals and their care were consistent with NIH guidelines (NIH Pub. No. 85–23, revised 1996) and approved by the Animal Care and Use Committee of Anhui Normal University under approval number #20110710. Tissue samples were collected from the tails (3–5 mm from the tip) of living turtles (AH1, AH2, AH3, Z2) at the sampling location using procedures that minimized pain. These tail samples were preserved in liquid nitrogen until use. The wounds were then sterilized with 75% ethanol, and dressed with gauze and absorbent cotton wool. The turtles were then immediately released into a local habitat. Tissue samples were collected from the muscles of cryopreserved samples.

### Sample collection and sequencing of duplicate CRs from the mt genome

Twenty specimens from each of the three turtle subspecies were collected from southern China and boundary areas adjacent to Vietnam and Myanmar (Table 1). These were chosen based on morphological characteristics described previously (*P.m. megacephalum* Gray 1831, *P.m. peguense* Gray 1870, *P.m. shiui* Ernst and McCord 1987) [19]–[22].

**Table 1:** Locations of sample collection.

Subspecies	Individual codes	Locations	Abbreviation
*P.m. megacephalum*	A1	Anhui, China	AH
	A2	Anhui, China	AH
	A3	Anhui, China	AH
	Z2	Zhejiang, China	ZJ
	FJ	Fujian, China	FJ
	X1	Fujian, China	FJ
	X4	Fujian, China	FJ
	X5	Fujian, China	FJ
	X7	Fujian, China	FJ
	X8	Fujian, China	FJ
	X9	Fujian, China	FJ
*P.m. shiui*	YN	Guangxi, China	VN
		(adjacent to Vietnam)	
	X6	Guangxi, China	VN
		(adjacent to Vietnam)	
	YP	Guangdong, China	GD
	Y1	Guangdong, China	GD
	Y2	Guangdong, China	GD
*P.m. peguense*	TP	Yunnan, China	MY
		(adjacent to Myanmar)	
	T1	Yunnan, China	MY
		(adjacent to Myanmar)	
	ZP	Guangxi, China	GX
	ZJ	Guangxi, China	GX

Genomic DNA extraction was performed using the phenol-chloroform method. Two pairs of primers were designed for each CR based on published mt genome sequences of *P. megacephalum* (DQ016387, DQ256377 [7], [8]) (Table 2). Outer primers were used to determine whether the duplicate CRs occurred in all individuals at the same locations as previously described [7], [8]. For CR1 whose position in the vertebrate mt genome is known, one outer primer (G17) was designed from the *cyt b* gene and the other (G20) was designed from the *12s rRNA* gene. A second pair of outer primers, G21 and G22, was designed to amplify the duplicate CR (CR2), located between the *nad1* and *nad2* genes. The amplification products of the outer primers were used as templates for the inner primers to ensure specificity. For CR1, inner primers, CR1S and CR1X, were located between *tRNA^Pro^* and *tRNA^Phe^* genes. The second pair of inner primers, CR2S and CR2X, was designed to amplify CR2 located between *tRNA^Pro^* and *tRNA^Gln^* genes. Long-template (LT) PCRs with outer primers were performed in a total volume of 25 µL containing 100 ng genomic DNA, 1.25 U LA Taq DNA polymerase (TaKaRa Co., Ltd, Dalian, China), 2.5 µL 10× PCR buffer, 3 mM MgCl_2_, 0.4 mM of each nucleotide and 0.1 µM of each primer. PCR cycling was performed as follows: 94°C for 1 min for pre-denaturation plus 38 cycles of 94°C for 10 s; 50–52°C for 5 min followed by an additional extension at 72°C for 10 min. PCR products were purified using an Axygen DNA Gel Extraction Kit. Then nested PCR was performed with inner primers and products of LT PCR as templates in a 25 µL reaction volume consisting of 100 ng template DNA, 1.25 U Taq DNA polymerase (TaKaRa Co., Ltd, Dalian, China), 2.5 µL 10× PCR buffer, 3 mM MgCl_2_, 0.4 mM of each nucleotide, and 0.1 µM of each primer. Nested PCR was performed as follows: 94°C for 1 min for pre-denaturation plus 94°C for 10 s and 50–52°C for 5 min for 30 cycles followed by an additional extension at 72°C for 10 min.

**Table 2:** Specific primers used to amplify duplicate CRs in *Platysternon megacephalum*.

Gene	Name	Sequences	Annealing temperature
CR1	G27	AGCAGCCTCCATCCTWTACTT	50–53°C
	G20	ATTGGCTACACCTTGACCTGAC	
	CR1S	AACCACATCATTCCGACCAC	50–53°C
	CR1X	CGGCTCCTTTGTCTAATAG	
CR2	Z22	TTTCTAACCGCACCTCAC	52–53°C
	G22	GTAGTTGGGTTTGGTTTAATCC	
	CR2S	CCCGAATAATAGACTCAACC	50–52°C
	CR2X	CGTGTGGGTTCATTAGTAAAGG	

Finally, the purified nested PCR products were cloned into a pM-18T vector (TaKaRa Co., Ltd, Dalian, China) and were transformed into *Escherichia coli* strain, DH5α. Plasmids isolated from single clones were sequenced in both directions to ensure the fidelity of each sequence using the ABI 3700 automated sequencer (Sangon Biotechnology Co., Ltd, Shanghai, China). All sequences were aligned using BioEdit 7.0 and ClustalX 1.8 software [23], [24]. Then all sequences were compared against sequences in GenBank using BLAST (http://blast.ncbi.nlm.nih.gov/Blast.cgi). Sequences with mutations were re-amplified using high-fidelity polymerase (TransGen Co., Ltd, Beijing, China), then re-cloned and re-sequenced. After ensuring their homology with published sequences by BLAST comparisons, all sequences were deposited in GenBank (Accession numbers KC476449-KC476496).

### Phylogenetic analysis

Positions of the duplicate control regions were identified from locations of tRNA genes (*tRNA^Pro^* and *tRNA^Phe^* for CR1; *tRNA^Pro^* and *tRNA^Gln^* for CR2) in the genome using tRNAscan-SE 1.21. (http://lowelab.ucsc.edu/tRNA_Scan-SE). Three functional regions including the terminal-associated sequence (TAS), central conserved domain (CD), and conserved sequence blocks (CSB), were identified in duplicate CRs through recognition of sequences similar to those found in other turtles [25], [26]. The mean genetic distances between CRs in all individuals were calculated from complete CR sequences using the Kimura-2 parameter model in the MEGA 4.1 program [27]. Variable number tandem repeats (VNTRs) have been reported to exist only at the 3′ end of the CR1 CSB region [7], [8]. Therefore, phylogenetic analyses were performed in CR sequences excluding the VNTRs, using the neighbor-joining (NJ) and maximum likelihood (ML) algorithms with PAUP*4.0 b10 [28]. Bayesian inference (BI) analyses were performed using MrBayes ver.3.2.1 on 2000,000 generations and 100 sampled generations [29]. The robustness of the NJ and ML trees was estimated using bootstrap proportion (BP) with 1000 replicates. Statistical support for the BI tree was evaluated with the posterior probability (PP). *Chrysemys picta* was designated as the outgroup in the phylogenetic analyses. To understand the evolutionary pattern in detail, unrooted phylogenetic trees were reconstructed using separate regions (TAS, CD, CSB, and VNTRs) based on BI analyses. Nucleotide substitution model (HKY85) was made with jModeltest Version 2.1.3 [30] using Akaike Information Criterion correction (AICc) prior to phylogenetic reconstruction.

### Recombination testing

Discordant evolutionary signals were detected when phylogenetic trees were reconstructed with different regions separately. These conflicting signals may be due to recombination of duplicate CRs. To further analyze these signals, four recombination tests for duplicate CRs were conducted: 1) Recombination detection program (RDP) [31], 2) Geneconv [32], 3) Maxchi [33] and 4) Chimaera [34]. Because of the heterologous nature of the VNTR lengths, it was necessary to collect sequences with same base pair lengths for alignment. Therefore, we chose only 4 individuals (AH2, YP, ZP, and TP) to represent the three subspecies. These analyses were performed using the RDP software with the parameters described previously [35]. Similar analyses were carried out for all individuals to make sure whether recombination is widespread in mtCRs of this turtle. All analyses were performed 3 times.

## Results

### Duplicate control regions in mtDNA of Platysternon megacephalum

**Figure 1:**
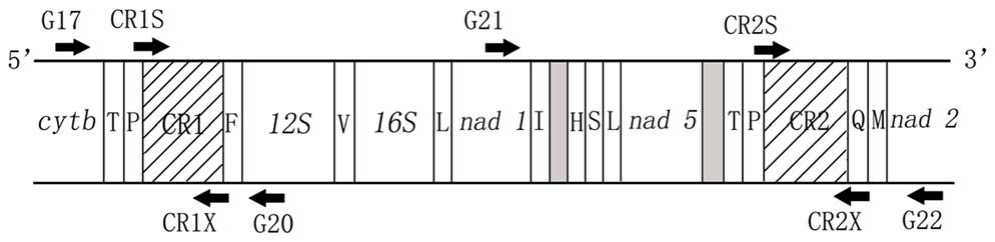
Locations of duplicate CRs and primers used for amplification. G17/G20 are outer primers for CR1; G21/G22 are outer primers for CR2. These primer pairs were used to determine the presence of CRs in the mtDNA of individual turtles. Inner primers, CR1S/CR1X and CR2S/CR2X, are designed to amplify specific duplicate CRs. The names of tRNA genes are shown using abbreviations of amino acids (T: *tRNA^Thr^*, P: *tRNA^Pro^*, F: *tRNA^Phe^*, V: *tRNA^Val^*, L: *tRNA^Leu^*, I: *tRNA^Ile^*, H: *tRNA^His^*, S: *tRNA^Ser^*, Q: *tRNA^Gln^*, M: *tRNA^Met^*). Gray boxes represent non-coding spaces between two genes. CR1 and CR2 are indicated with slashes.

PCR of this turtle's mtDNA using outer primers amplified duplicate regions that contained both *cyt b* and *12s rRNA* for CR1 and both *nad1* and *nad2* for CR2. This showed that duplicate CRs were present in all individuals collected. The positions of duplicate CRs as described by Peng *et al.* and Parham *et al.* [7], [8] are shown in Fig. 1. The length of CR1 ranged from 1,222–1,608 bp and CR2 ranged from 1,128–1,420 bp. Because of the absence of VNTRs in CR2, sequences in the 3′ end of CR1 and CR2 could not be aligned properly. However, approximately 800 bp of the 5′ end of CR1 and CR2 was aligned properly with few indels, as descried previously [7], [8].

### Structures of duplicate CRs

**Figure 2:**
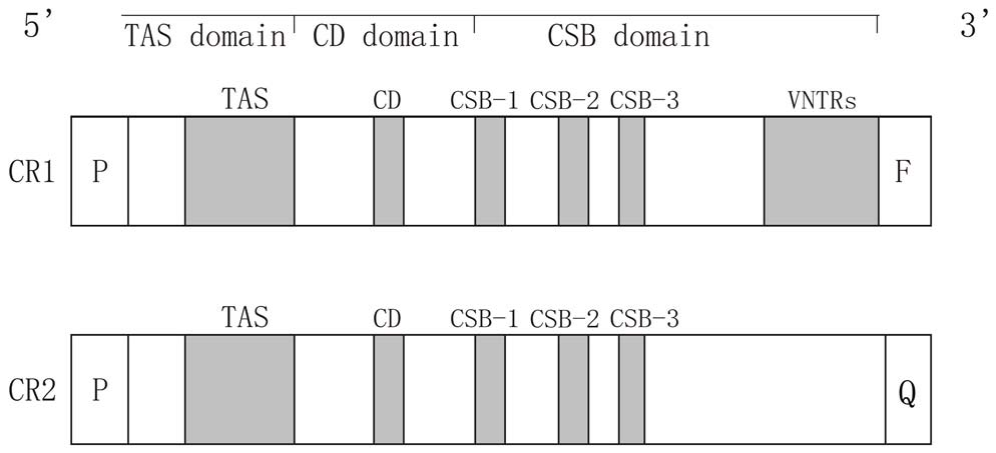
Structures of duplicate CRs in individuals. Three functional regions, TAS, CD, and CSB, were detected in both CRs in all individuals. The core sequences of these regions were found to be identical in CR1 and CR2. Variable numbers of tandem repeats (VNTRs) were detected only in CR1.

Three conserved functional sections including the TAS, CD, and CSB domains were detected in both CR1 and CR2 in all individuals (Fig. 2). The TAS domain is in general affiliated with termination of the H-strand by forming a very stable hairpin structure between its core sequence (5′-TACAT-3′) and the reversed complementary sequence (5′-ATGTA-3′). The central conserved domain (CD) is located downstream of the TAS domain and is 459 bp long. It is characterized by the conserved sequence 5′-AATCACGAGAGATAAGCAA-3′. All three conserved sequences, CSB-1, CSB-2, and CSB-3 were found in the 5′ end of the CSB domain and were recognized by the presence of 5′-TTAATGCTTGTTAGACATA-3′ for CSB-1, 5′-TTAAACCCCCCTACCCCCC-3′ for CSB-2 and 5′-TCGTCAAACCCCTAAATCC-3′ for CSB-3 [25], [26], [36]. All core sequences were identical in all duplicate CRs, and there was no variation among the 20 individuals.

**Figure 3:**
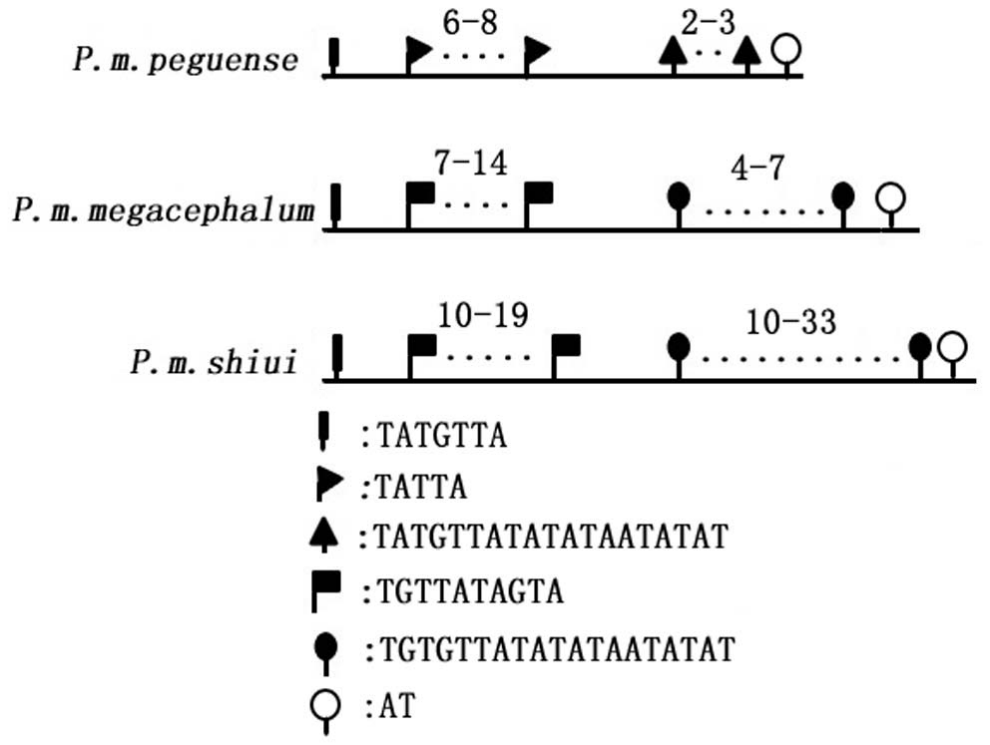
Heterology of VNTRs in different subspecies. The line at the bottom of the image indicates the lengths of VNTRs. The numbers above the dots represent the copy numbers of motifs.

In the 3′ end of CSB, VNTRs with heterology in length and motif were detected in the mtDNA of this turtle as previously reported [7], [8]. In all individuals, CR2 had a specific sequence that was 411 bp in length after the 3′ end of CSB. All these sequences were aligned with few indels and only 60 bp were variable in different individuals. However, significant differences in CR1 sequences were detected with respect to both length and motif. The length of VNTRs varied from 77 bp to 786 bp leading to length heterology of mtDNA. The first repeat unit, TATGTTA, was detected in all individuals, and the AT motif was the last repeat motif in all individuals. Although the beginning and ending units were identical in all subspecies, the remaining repeat units contained subspecies-specific heterology in both length and motif sequence. In CR1 within mtDNA of subspecies, *P.m. peguence*, the repeat motifs were relatively simple and contained TATTA (repeated 6–8 times) and TATGTTATATATAATATAT (repeated 2–3 times). However, the repeat units in *P.m. megacephalum* and *P.m. shiui* had similar motifs (TGTTATAGTA and TGTGTTATATATAATATAT) but differed only in the number of repeats (Fig. 3).

### Phylogenetic analyses of duplicate CRs

The genetic distances calculated by MEGA 4.1 software using the complete sequences of duplicate CRs (Table 3) suggested that orthologous copies were more similar than paralogous copies. Because of the absence of VNTRs in the 3′ end of the CSB domain, the genetic distance of these regions showed significantly more independent evolutionary signals with dDup  = 36.4%, while dInt was only 0.7%.

**Table 3:** Mean genetic distance calculated using MEGA 4.1 software and different parts of CRs.

		TAS		CD		CSB		VNTRs		TOTAL^1^	
		dInt^2^	dDup^3^	dInt^2^	dDup^3^	dInt^2^	dDup^3^	dInt^2^	dDup^3^	dInt^2^	dDup^3^
*P.m.*	CR1	0.020	0.016	0.005	0.007	0.001	0.001	0.010	0.362	0.007	0.048
*megachephalum*	CR2	0.010		0.008		0.000		0.009		0.007	
*P.m .shiui*	CR1	0.008	0.052	0.011	0.011	0.005	0.003	0.004	0.378	0.008	0.052
	CR2	0.013		0.010		0.000		0.009		0.009	
*P.m. peguense*	CR1	0.008	0.064	0.002	0.005	0.003	0.002	0.005	0.352	0.004	0.043
	CR2	0.117		0.008		0.000		0.005		0.020	

1 Total: Genetic distances calculated using complete sequences.

2 dInt: Genetic distances of orthologous copies from different individuals.

3 dDup: Genetic distances of paralogous copies from duplicate CRs within the same individual.

**Figure 4:**
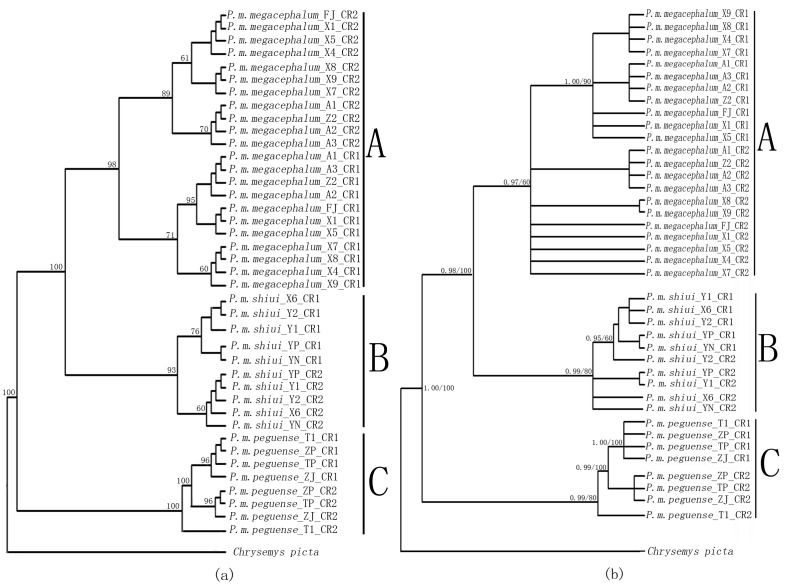
Phylogenetic trees reconstructed using (a) neighbor-joining, (b) maximum likelihood and Bayesian methods. Three clades were grouped. These contained the three subspecies of *Platysternon megacephalum*: (A) *P.m. megacephalum*, (B) *P.m. shiui*, and (C) *P.m. peguense*.

Phylogenetic trees generated using the NJ, ML, and BI methods showed consensus in almost all the nodes and revealed three major clades (Fig. 4). These clades were consistent with the subspecies taxonomy with clades A and B representing *P.m. megacephalum* and *P.m. shiui*, respectively. These two subspecies were more phylogenetically familiar than the subspecies *P.m. peguense* which is represented by clade C in the phylogenetic trees. In each clade, the orthology of CR1 or CR2 from different individuals were grouped together. Each CR was distinct from the placement of paralogous copies within same individuals. Although these phylogenetic trees share similar topology in general, differences did exist (Fig. 4). For example, in NJ trees, CR1 were distinguished from CR2 in all clades without exceptions although some CR2 in clade A from the ML and BI trees showed parallel branching. CR1 and CR2 from the same individuals were always separate from each other and did not form clusters.

**Figure 5:**
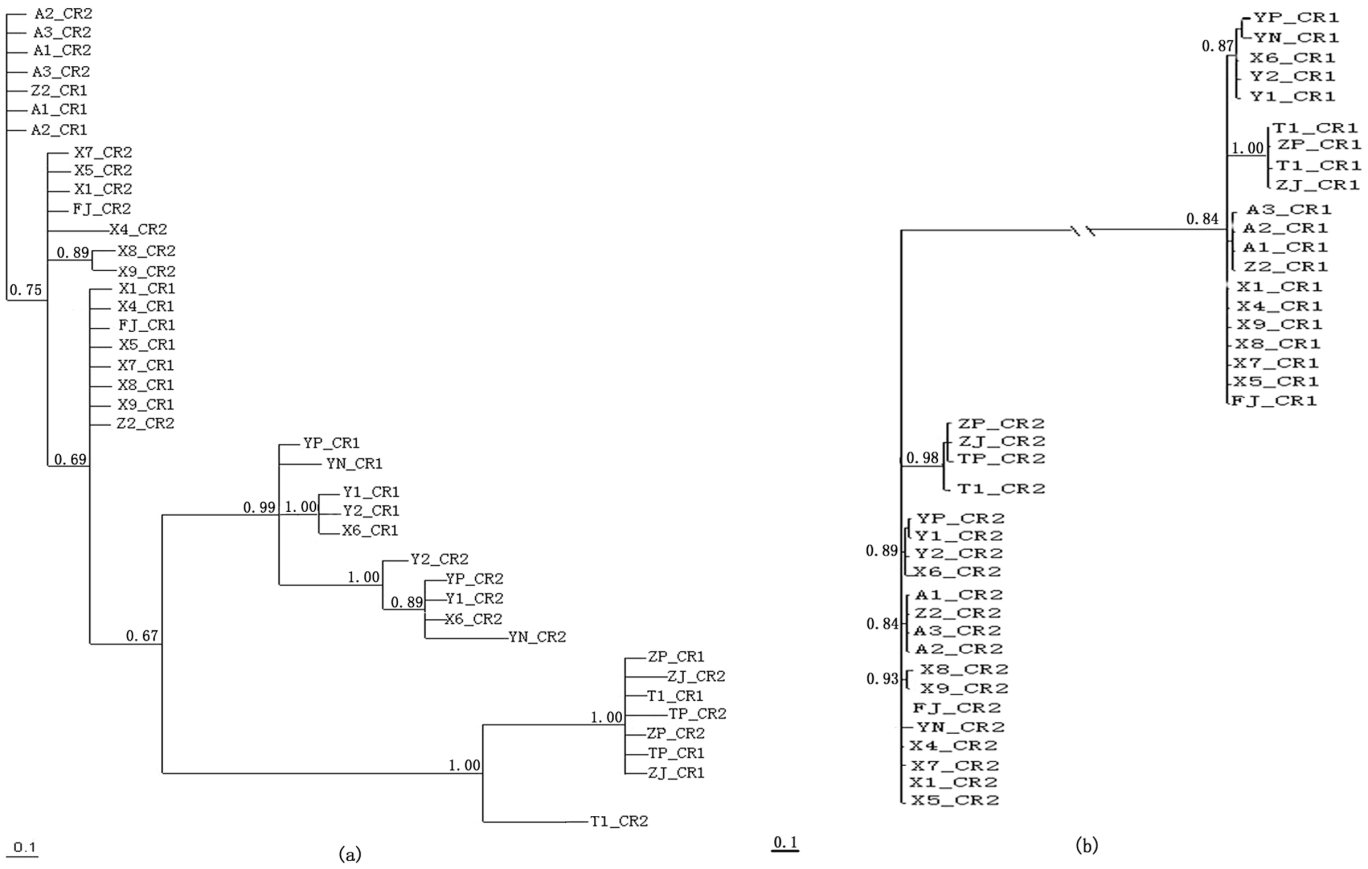
Phylogenetic trees reconstructed using (a) TAS, CD, and CSB domains and (b) VNTRs. All three phylogenetic trees constructed separately based on TAS, CD, and CSB shared similar topology and therefore only one tree is presented here.

Conflicting evolutionary signals were observed when phylogenetic trees were reconstructed separately using the divided regions (Fig. 5). Phylogenetic trees of TAS, CD, and CSB sequences shared the same topologies, showing that paralogous copies from the same individuals always clustered together rather than with orthologous copies from different individuals. However, the opposite was observed in the phylogenetic tree built using VNTRs. Orthologous copies were more closely related to their counterparts from different individuals rather than to paralogous copies from the same individual. In addition, the divergence of duplicate CRs occurs earlier than the differentiation of subspecies, as shown in the VNTRs tree (Fig. 5).

**Figure 6:**
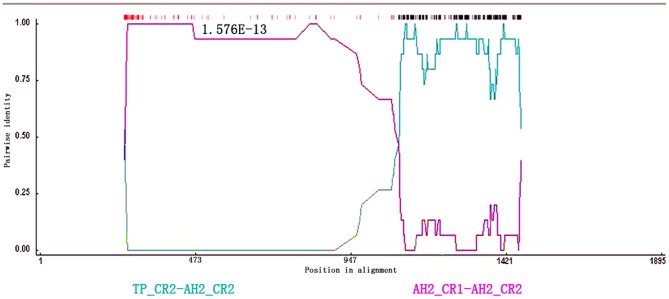
Assessment of recombination in duplicate CRs detected by RDP software in an individual, AH2. Green shade indicates pairwise identity between orthologous CR2 genes in individuals, TP and AH2. Purple shade depicts pairwise identity between paralogous CRs from AH2. Similar analysis was performed for all individuals.

To explain the presence of discordant signals in the phylogenetic trees constructed separately using the three different regions, multiple recombination points were investigated using the RDP software (Fig. 6). In total, 4 recombination events were detected in all tested individuals. For example, CR1, from individual AH2, displayed very high pairwise identity in common with its paralogous copy CR2, but very low pairwise identity compared to CR2 from the individual, TP. However, recombination events in AH2 were indicated by all four analyses (RDP, *P* = 1.576×10^−13^, MaxChi, *P* = 5.035×10^−14^, Chimaera, *P* = 3.915×10^−13^, Geneconv, *P* = 1.622×10^−10^). Similar recombination signal were also detected in other individuals (not shown) and the positions of recombination breakpoints that occurred in duplicate CR, without exception, were about 290 bp and 1080 bp.

## Discussion

### Evolutionary pattern of duplicate CRs in P. megacephalum

The mt gene order is highly conserved in Testudines [25]. Thus far, only two mt genomic rearrangements have been reported in two species, the pancake turtle and the Asian big-headed turtle [7], [8], [18]. However, whether duplicate CRs evolved concertedly as they did in other deuterostomes is unclear.

The mean genetic distances between duplicate CRs indicated that orthologous copies (dDup_CR1 = 0.63%; dDup_CR2 = 1.2%) were more similar to each other than to their paralogous counterparts (dDup = 4.8%) within each subspecies (Table 3). In addition, phylogenetic trees reflected a closer relationship among single CRs in different individuals than between two copies of CRs within same individual of a subspecies. In this way, orthologous CRs from different individuals were genetically more similar than paralogous CRs from the same individual within any subspecies clade. This evolutionary pattern indicated independent evolution of duplicate CRs within every subspecies of *P. megacephalum*; that is CRs evolution was not affected by each other. However, it appears that duplicate CRs evolved concertedly among different subspecies, considering that both CR1 and CR2 from each subspecies were more similar or more closely related to each other than either of their own counterparts from other subspecies. In addition, duplicate CRs divided with each other much earlier than the differentiation of subspecies as shown in phylogenetic trees constructed using VNTRs (Fig. 5b). Based on these findings, the following evolutionary scenarios were deduced: 1) originally, the ancestors of Asian big-headed turtles had only one CR in their mtDNA. After a mutation event, the CR could have duplicated, which may have offered some advantages to this turtle. Because of this, the mtDNA with duplicate CRs may have been preserved during reproduction. 2) mtDNA with duplicate CRs may have been acquired by subspecies during the separation process. This could be another way the duplicate CRs could have evolved among different subspecies in a concerted manner. The duplicate CRs could play different roles in the replication of mtDNA so that the duplicate CRs may have evolved independently within any subspecies. Whether different functions are performed by the duplicate CRs requires further investigation at the transcriptome and proteome levels.

### Possible role of recombination in the evolutionary pattern of duplicate CRs

The data collected here generated specific questions such as how did the ancestral mtDNA of Asian big-headed turtles obtain duplicate CRs. It has been suggested that tandem duplication, dimerization, or illegitimate recombination could have played a role in that process [1]. Tandem duplication is caused by errors in replication, such as slipped strand mispairing, over-running the terminal signal of the mt genome, and initiation of replication from a secondary structure [1], [37]–[40]. Two linearized monomeric mt genomes could have dimerized through head to tail joining to form a large circular mt genome containing two copies of each gene, including the CR. Finally, illegitimate recombination may have given rise to tandem duplication when a section mt genome was spliced out and introduced into another region within the same mitochondrion [41]. Based on our data from this study, duplicate CRs in mtDNA of *P. megacephalum* could have originated by heterologous recombination between two divergent CRs.

The discordant signals revealed by phylogenetic trees using different CR components suggested that recombination could have influenced the evolution of duplicate CRs in mtDNA of *P. megacephalum*. Previously, Abbott proposed a contrasting pattern of CR evolution in *Thalassarche albatrosses*: a section containing two copies could evolve independently and another section could evolve in a concerted fashion [16], which was indicated by a specific point of recombination found in F1. A recent study on seabirds (Sulidae) suggested that the 5′ end of CRs evolved independently while the duplicate CRs, as a whole sequence, evolved concertedly [15]. Further, Morris-Pocock *et al.* proposed that differences in CR evolution could be difficult to resolve without testing multiple recombination points, as described by Abbott [16], [17].

Moreover, four recombination points were tested using RDP software with specific attention to distinct signals for the evolution of duplicate CRs. These data showed the break points of recombination at 290 bp and 1,080 bp upstream of the TAS domain or the VNTRs, respectively. Sequencing of the complete mtDNA of two Philippine hornbill species (Aves: Bucerotidae) showed a replication fork barrier (RFB) downstream of ETAS [42]. During each replication cycle, the 3′ end of the nascent L-strand was suspected to remain free at the RFB region until replication restarted. During the relatively long period of exposure, the free strands could be easily exchanged, leading to a high rate of recombination [42].

Heterologous sequences are considered essential to mtDNA recombination [43]. In the mtDNA of flounder, *Platichthys flesus*, two heterogeneous patterns (C and T arrays) were found in the VNTRs motif all 168 individuals analyzed [44]. Most individuals had only pure C or T arrays, but one individual had a compound CT array. This is a direct evidence of recombination between mtDNA in the flounder. Other evidence of heterologous recombination of mtDNA has been reported in various animals [45]–[48]. Our data showed that the sequences at the 3′ end of the CSB domain of CR1 and CR2 differed significantly. Specifically, the VNTRs in the CR1 of different subspecies were heterologous with respect to the length and motif sequences. These findings may explain how recombination of duplicate CRs was induced in the **mtDNA of P. megacephalum**

The data from this study facilitates the hypothesis that CR1 and CR2 could have originated from different ancient individuals with only one CR, and then crossing over may have occurred between heterologous mt genomes, allowing the insertion of several genes containing CRs into other genomes through an occasional paternal linkage [49]. Such genetic variation could be maintained in the mt genome of *P. megacephalum* over time due to potential advantages by maintaining duplicate CRs [50], [51]. In addition, duplicate CRs may contribute to different functions, so that evolutionary pressure that could have existed earlier during evolution to change the CRs may have diminished. This could be a plausible way the CRs would have evolved independently within each subspecies after the divergence of subspecies and accumulated mutations independently over time.

Another question raised by our findings is what maintains over time the main functions of CRs, such as initiation of replication and transcription of mtDNA during the independent evolution of duplicate CRs. It has been proposed that intra-molecular recombination may occur through gene conversion when mtDNA has three strands [14]. The nascent H-strand of one CR then recombines with homologous parental strands of another CR, leading to the homogenization of both CRs. Our data show that homogenization of the TAS, CD, and CSB domains could have occurred via recombination upstream of TAS and VNTRs. During every replication cycle, a nascent H-strand with one CR (CR1 or CR2) may have crossed over to the parental strand of another CR at the break points. Then homogenization may have taken place in the TAS, CD, or CSB domains, leading to concerted evolution of these two areas while allowing independent evolution of TAS and VNTRs. Some essential elements, such as the termination and initiation sites of the H-strand, were included in TAS, CD, and CSB [3]. In this way, sequence homogenization of these three regions would have been critical to ensure the functions of these CRs during periods of independent evolution.

## Conclusion

This is the first report describing the evolution of duplicate CRs within the Testudines mt genome. Our findings show that in *P. megacephalum* duplicate CRs evolved independently within subspecies but concertedly across subspecies. Sequence homogenization in duplicated CRs may have resulted from recombination to ensure their function during evolution.
